# Ten-year Survival Rate of Cement- and Screw-retained Restorations on Bone-level Dental Implants in Grafted and Non-grafted Sites: A Retrospective Study

**DOI:** 10.3290/j.ohpd.b2082139

**Published:** 2021-09-30

**Authors:** Bandar Awadh Alresheedi, Saad Obaid Alazmi, Faris Jaser Almutairi

**Affiliations:** a Assistant Professor, College of Dentistry, Qassim University, Qassim, Saudi Arabia. Study design, wrote the results section, read and revised the manuscript.; b Assistant Professor, College of Dentistry, Qassim University, Qassim, Saudi Arabia. Study design, clinical and radiographic examinations, read and revised the manuscript.; c Assistant Professor, College of Dentistry, Qassim University, Qassim, Saudi Arabia. Clinical and radiographic examinations, wrote the introduction and methods section, read and revised the manuscript.

**Keywords:** cement-retained, dental implant, inflammation, screw-retained, survival

## Abstract

**Purpose:**

The aim of the present 10-year follow-up study was to assess the survival rate of cement- and screw-retained restorations on dental implants placed in grafted sites.

**Materials and Methods:**

Patients with cement- (group 1) and screw-retained (group 2) restorations on implants placed in grafted sites and patients with cement- (group 3) and screw-retained (group 4) restorations on implants placed in non-grafted sites were included. Demographic data was recorded using a questionnaire, and information regarding implant dimensions, surface characteristics, insertion torque, type of bone graft used, jaw location and duration of implants in function was retrieved from patients’ records. These patients were evaluated for peri-implant crestal bone loss (CBL), probing depth (PD), modified plaque index (mPI), and modified bleeding on probing (mBOP). p < 0.05 was considered statistically significant.

**Results:**

Eighty-eight partially edentulous individuals (n = 22 in each group) were included. The mean ages of individuals in all groups were comparable in all groups. In each patient, 1 bone-level platform-switched dental implant with moderately rough surfaces was placed using an insertion torque of 30–35 Ncm. In all groups, the length and diameter of implants ranged between 11–14 mm and 4.1–5 mm, respectively. There was no statistically significant difference in mPI, mBoP, PD, and mesial and distal CBR around implants in any of the groups.

**Conclusion:**

Bone-level implants restored with cement and screw-retained restorations can possess a stable clinicoradiographic status and remain functional in grafted and non-grafted sites, provided strict domestic oral hygiene measures are adopted and routine dental prophylaxis is carried out by oral healthcare providers.

The choice of implant prosthesis retention (cement- vs screw-based retention) continues to be subject of debate in clinical implant dentistry and related research.^2,22^ Traditional clinical and laboratory-based techniques are used for the fabrication of cement-retained restorations, which makes this form of retention less challenging than screw-retained implant restorations.^22^ Moreover, occlusal contacts are easily stabilised using cement-retained restorations, as occlusal screw-access holes are absent in this form of implant prosthesis retention.^21,25^ In a study by Hameed et al,^15^ screw-retained implant prostheses demonstrated statistically significantly greater loss of crestal bone compared with cement-retained implant prosthesis. In contrast, Amri et al^2^ found that the mode of prosthesis retention does not influence the peri-implant clinical (modified bleeding on probing [mBoP], probing depth [PD]) or radiographic (crestal bone loss [CBL]) inflammatory parameters. Nevertheless, it has also been proposed that accumulation of residual cement particularly at the restoration margins is a causative factor in the initiation of peri-implant soft tissue inflammation.^27,31^ This is often observed in situations where the restoration margins extend at least 3 mm subgingivally.^27^ It has been reported that cement-retained fixed implant-supported prostheses demonstrate less CBL than screw-retained fixed implant-supported prostheses.^22^ However, there are no clinical studies that have compared the survival rates of cement- and screw-retained restorations on dental implants placed in grafted sites.

Alveolar bone grafting (ABG) includes procedures employed to generate and direct bone formation using barriers at sites with insufficient volumes of bone for biological and aesthetic success.^29,33^ In clinical implant dentistry and related research, ABG is often performed prior to or during implant placement in sites with deficient bone or resorbed osseous ridges. The rationale of ABG is to prevent infiltration of undesirable epithelial and connective tissue cells from proliferating into the grafted site, allowing osteoprogenitor and other bone forming cells to repopulate for neo-osteogenesis.^11,13,26^ Guided bone regeneration (GBR) has been used clinically for a variety of indications, including peri-implant bone augmentation,^7,10^ extraction socket bone defects,^9^ ridge augmentations^17^ and periodontal defects.^8^ Criteria for barriers used (e.g. membranes and other devices) in GBR include cell occlusion, biocompatibility, space creation, tissue integration and clinical workability.^14,16^ The authors hypothesise that there is no difference in the mBoP, PD and CBL around cement- and screw-retained dental implants placed in grafted and non-grafted sites.

The aim of the present retrospective study was to assess the survival rate of cement- and screw-retained restorations on bone-level dental implants in grafted and non-grafted sites at 10 years of follow-up.

## Materials and Methods

### Ethical Approval

Ethical approval was obtained from the ethics research committee of the Centre for Specialist Dental Practice and Clinical Research, Saudi Arabia (UDCRC/025-0054). Guidelines recognised by the Helsinki- Declaration for experimentation involving humans were followed. All participants were obliged to read and sign a consent form. All participants reserved the right to withdraw at any phase without penalty. All participants were given written information sheets about oral hygiene maintenance.

### Inclusion and Exclusion Criteria

The inclusion criteria were: (a) patients with cement-retained single-unit restorations on dental implants placed in grafted sites; (b) screw-retained single-unit restorations on dental implants placed in grafted sites; (c) patients with cement-retained single-unit restorations on dental implants placed in non-grafted sites; (d) patients with screw-retained restorations on dental implants placed in non-grafted sites. The exclusion criteria were: (a) use of adjuvant osseous augmentation techniques such as growth factors; (b) tobacco smokers and smokeless tobacco chewers; (c) patients with self-reported systemic diseases such as diabetes mellitus; (d) patients with a history of periodontitis; (e) refusal to sign the consent form.

### Participants and Groups

Participants were divided into 4 groups: 1: patients with cement-retained single-unit restorations on dental implants placed in grafted sites; 2: patients with single-unit screw-retained restorations on dental implants placed in grafted sites; 3: patients with cement-retained single-unit restorations on dental implants placed in non-grafted sites; 4: patients with screw-retained restorations on dental implants placed in non-grafted sites.

### Invitation to the Present Study

An invitation letter that explained the objectives of the present study in simple English and Arabic was sent by postal mail to individuals who had undergone dental implant therapy at least a decade ago. This information was retrieved from patients’ dental records. A total of 200 letters (100 to patients who had implants placed in grafted and 100 to those with non-grafted sites). The response rate was 68%.

### Questionnaire

Information related to age, gender, duration of cement- and screw-retained implants in function, jaw location of implants, and oral hygiene (brushing and flossing) was collected using a questionnaire. This questionnaire was administered to all patients by a trained investigator. Patients’ dental records were also assessed to determine the features of implants (dimensions, surface characteristics, timing of implant placement and cement used for restoration) that were placed. Information about the type of osseous graft used (allograft, xenograft or alloplastic) was recorded from the patients’ dental records.

### Clinical and Radiological Evaluation

In this study, CBL was calculated as the perpendicular distance from 2 mm under the abutment-implant junction up to the top of the alveolar crest. This calculation was done on digital bitewing radiographs, which were taken using the long-cone paralleling technique.^13^ A positioner (X-ray Holders, KerrHawe; Bioggio, Switzerland) was positioned on 30.5- x 40.5-mm film (Kodak-Ultraspeed size II Dental-Film, Kodak; Rochester, NY, USA) parallel to the long axis of the implant and perpendicular to the X-ray cone.^12^ The radiographs were also assessed for evidence of excess cement accumulation in the subgingival region. This assessment was done by one investigator who had an intra-examiner score of 0.92. One investigator (Kappa score 0.94) measured the mPI, mBoP and PD around implants in all groups. The peri-implant sites (3 buccal and 3 palatal/lingual) were gently probed, and any bleeding was recorded. The mBoP was recorded as a percentage of sites per implant that bled upon probing using the formula: (sites that bled/ 6 sites) x 100. The PD was measured in millimeters using a graded plastic probe.

### Statistical Analysis

A software package was used to perform the statistical comparisons among the study groups (SPSS v 20; Chicago, IL, USA). Data normality was determined using the Shapiro-Wilk test. Group comparisons were performed using one-way ANOVA. For multiple comparisons, Bonferroni’s post-hoc adjustment test was carried out. p-values below 0.05 were considered statistically significant. The sample size was estimated based upon the results of a pilot investigation. It was estimated that inclusion of at least 22 individuals per group would be necessary to give the study a power of 90% with an alpha error of 0.05.

## Results

### General Characteristics

Eighty-eight partially edentulous individuals (n = 22 per group) were included. In all groups, most of the participants were male. The mean ages of individuals in groups 1, 2, 3 and 4 were 57.2 ±2.9, 56.4 ± 2.1, 55.7 ± 1.3 and 56.1 ± 2.2 years, respectively. In each patient, one bone-level platform-switched dental implant with moderately rough surfaces was placed using an insertion torque of 30-35 Ncm. In all groups, the length and diameter of implants ranged between 11-14 mm and 4.1-5 mm, respectively. In groups 1, 2, 3 and 4, implants were in function for 10.3 ± 0.2, 10.5 ± 0.4, 10.4 ± 0.4 and 10.5 ± 0.3 years, respectively. Toothbrushing twice daily was reported by 80%, 73.3%, 80% and 86.7% of individuals in groups 1, 2, 3 and 4, respectively. Full-mouth interdental flossing twice daily was reported by 26.7%, 40%, 46.7% and 33.3% individuals in groups 1, 2, 3 and 4, respectively. Forty percent, 46.7%, 53.3% and 40% individuals in groups 1, 2, 3 and 4, respectively, reported that they visited their oral healthcare providers semi-annually for routine check-ups (Table 1).

**Table 1:** Characteristics of the study cohort

Parameters	Group 1	Group 2	Group 3	Group 4
Patients (n)	22	22	22	22
Age in years (mean ± SD)	57.2 ± 2.9	56.4 ± 2.1	55.7 ± 1.3	56.1 ± 2.2
Gender (M:F)	12:2	12:3	13:2	14:1
Jaw location (mandible:maxilla)*	8:7	9:6	9:6	8:7
Duration of implants in function	10.3 ± 0.2 years	10.5 ± 0.4 years	10.4 ± 0.4 years	10.5 ± 0.3 years
Daily toothbrushing				
Once daily	3 (20%)	4 (26.7%)	3 (20%)	2 (13.3%)
Twice daily	12 (80%)	11 (73.3%)	12 (80%)	13 (86.7%)
Flossing				
Once daily	11 (73.3%)	9 (60%)	8 (53.3%)	10 (66.7%)
Twice daily	4 (26.7%)	6 (40%)	7 (46.7%)	5 (33.3%)
Visits to oral healthcare provider				
Annually	9 (60%)	8 (53.3%)	7 (46.7%)	9 (60%)
Semi-annually	6 (40%)	7 (46.7%)	8 (53.3%)	6 (40%)

Group 1: patients with cement-retained single-unit restorations on dental implants placed in grafted sites; group 2: patients with single unit screw-retained restorations on dental implants placed in grafted sites; group 3: patients with cement-retained single-unit restorations on dental implants placed in non-grafted sites; group 4: patients with screw-retained restorations on dental implants placed in non-grafted sites. *All implants were placed in the regions of missing premolars or molars.

### Grafting and Implant-related Characteristics

In groups 1 and 2, particulate bovine xenografts and collagen membranes were used for osseous augmentation at the recipient sites. Bone grafting was done for horizontal bone augmentation in healed extraction sites. In groups 1 and 2, osseous grafting was performed 3.6 ± 0.3 and 3.5 ± 0.2 months, respectively, prior to implant placement. All implants were platform switched, had moderately rough surfaces, and their diameters and lengths ranging between 4.1-4.8 and 11-13, mm, respectively.

**Fig 1:**
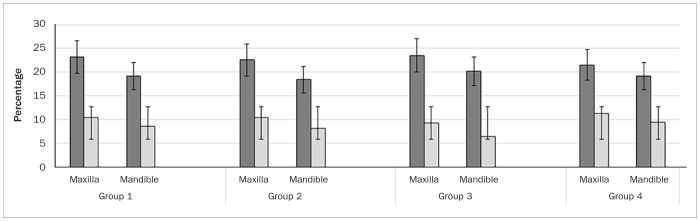
Comparison of modified plaque (dark grey bars) and bleeding (light grey bars) indices around implants in the study groups.

**Fig 2:**
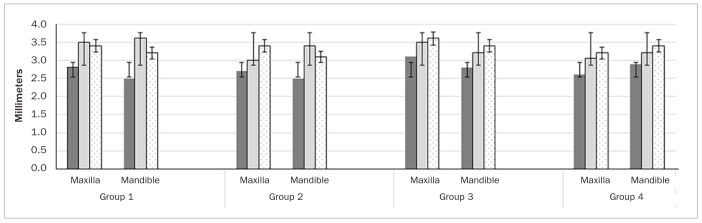
Comparison of probing depth (dark grey bars), mesial (light grey bars) and distal (dotted bars) crestal bone loss around implants in the study groups.

In each group, one dental implant (15 implants per group) was placed in the region of missing premolars or molars. In all groups, implants were delay loaded, placed at bone level using an insertion torque ranging between 30-35 Ncm by an experienced oral surgeon. In groups 1, 2, 3 and 4, implant loading was performed at 3.3 ± 0.2, 3.3 ± 0.2, 3.1 ± 0.08 and 3.2 ± 0.1 months, respectively. In each group, nearly 50% of implants were placed in the maxilla. There was no statistically significant difference in mPI, mBoP, PD, and mesial and distal CBL around implants in any of the groups. In all groups, none of the implants were lost up to 10 years of follow-up (Table 2). There was no statistically significant difference in mPI, mBoP, PD, and mesial and distal CBL around implants placed in the maxilla and mandible among participants in all groups (Figs 1 and 2).

**Table 2:** Peri-implant clinicoradiographic parameters in the study group

Parameters Mean (range)	Group 1	Group 2	Group 3	Group 4
Number of implants	22	22	22	22
Modified plaque index	18.6% (15.5–21.6%)	20.4 (19.6–22.7%)	21.2 (18.5–27.4%)	20.4 (17.7–21.4%)
Modified bleeding on probing	8.9 (0–10.1%)	9.5 (0–11.4%)	6.5% (0–8.3%)	10.2% (2.4–12.2%)
Probing depth	2.4 (2–3.2 mm)	2.3 (1.8–2.5 mm)	2.5 (2–3.3 mm)	2.4 (1.8–2.7 mm)
Crestal bone loss (mesial)	3.7 (3–4.4 mm)	3.2 (2.8–3.7 mm)	3.3 (3–3.4 mm)	3.2 (2.8–3.5 mm)
Crestal bone loss (distal)	3.5 (3–4.8 mm)	3.3 (2.7–3.6 mm)	3.5 (3.2–3.6 mm)	3.3 (2.9–3.7 mm)
Implant loss	0	0	0	0

## Discussion

The result of the present 10-year follow-up observational study showed no statistically significant difference in the peri-implant soft tissue inflammatory parameters (mPI, mBoP and PD) and CBL among patients that underwent implant therapy in grafted and non-grafted sites. These results indicate that dental implants can osseointegrate and remain stable for prolonged durations in grafted and non-grafted sites. A number of factors may have been contributory in this regard. Firstly, a factor that seems to have played a critical role in the success and survival of cement- and screw-retained restorations on dental implants placed in grafted and non-grafted sites is that all participants followed routine oral hygiene maintenance protocols. In the present study, at least 70% of the individuals in all groups reported brushing twice daily. Although interdental flossing twice daily was less often practiced by all patients, at least 50% individuals in all groups flossed once daily. Furthermore, it is important to mention that nearly half of the participants in each group visited their oral healthcare providers semi-annually (most likely every 6 months) for routine check-ups. It is speculated that during the routine dental visits, these individuals underwent full-mouth plaque and/or calculus removal using traditional prophylactic methods such as ultrasonic scaling. This suggests that the daily oral hygiene maintenance protocols adopted by the patients, in addition to professional evaluation and prophylaxis by oral healthcare providers, played a role in maintaining clinicoradiographic stability as well as the survival of cement- and screw-retained dental implants placed in grafted and non-grafted sites. The literature contains abundant evidence that a high educational status is directly associated with a superior oral health status.^19^ It is therefore speculated that all participants included in the present investigation were educated enough to comprehend the significance of oral hygiene maintenance, which leads to the long-term survival of dental implants without complications. This also suggests that patient education and routine dental follow-ups/prophylaxis are critical for maintaining a healthy periodontal and peri-implant soft tissue status and crestal bone levels. The present authors agree with the study by Tran et al,^32^ in which the authors proposed that a lack of professional maintenance is statistically significantly associated with implant failure.

It has been reported that operators’ clinical experience in terms of the number of implants they have placed plays a role in the stability and survival of dental implants.^28,30^ In the present study, evaluation of patients’ records revealed that all implants were placed and loaded by trained and experienced clinicians. However, by no means does this statement suggest that the failure rate of implants is higher when implants are placed and/or loaded by clinicians with limited clinical experience in the field of implant dentistry. According to Malmström et al,^24^ general-dentistry residents can achieve competence in the surgical as well as prosthetic phases of implant therapy while enrolled in an advanced general-dentistry program.

In the present study, stringent eligibility criteria were imposed on the selection of study participants. Tobacco smokers and immunosuppressed individuals were excluded. It is well known that habitual use of tobacco products (such as cigarette and waterpipe smoking) enhances soft tissue inflammation and augments CBL, thereby predisposing vulnerable patients to peri-implant diseases (peri-implant mucositis and peri-implantitis).^1,3,4,19,32^ Likewise, a state of persistent hyperglycemia, which is a common manifestation among patients with poorly controlled DM, is also a risk-factor for periodontal and peri-implant diseases.^5,6,20^ Moreover, smoking and impaired glycemic levels are also risk factors for early graft failure in susceptible patients.^23,32^ It is also important to note that the thickness of the keratinized mucosa (KM) was not measured in the present study. Based upon the present results, it is speculated that in the long term (at least 10 years of follow-up), there is no difference in the thickness of KM around implants placed in grafted and non-grafted sites. Further studies are needed to assess the influence of glycemic control and tobacco-smoking cessation on the survival of dental implants in grafted and non-grafted sites.

## Conclusion

Dental implants can demonstrate stable clinicoradiographic status and remain functional in grafted and non-grafted sites, provided strict domestic oral hygiene measures are kept and routine dental prophylaxis is carried out by oral healthcare providers.
